# Religious service attendance, divorce, and remarriage among U.S. nurses in mid and late life

**DOI:** 10.1371/journal.pone.0207778

**Published:** 2018-12-03

**Authors:** Shanshan Li, Laura D. Kubzansky, Tyler J. VanderWeele

**Affiliations:** 1 Slone Epidemiology Center, Boston University, Boston, Massachusetts, United States of America; 2 Department of Social and Behavioral Sciences, Harvard T. H. Chan School of Public Health, Boston, Massachusetts, United States of America; 3 Department of Epidemiology, Harvard T. H. Chan School of Public Health, Boston, Massachusetts, United States of America; 4 Department of Biostatistics, Harvard T. H. Chan School of Public Health, Boston, Massachusetts, United States of America; Coventry University, UNITED KINGDOM

## Abstract

Prior research has suggested religious participation can promote marital satisfaction and stability. However, current literature has mainly focused on early life divorce, and used cross-sectional data, leaving open the question of the directionality of effects. We evaluated the prospective associations between service attendance and marital stability in mid and late life considering either 1) divorce or separation; or 2) remarriage, as separate outcomes. Data were drawn from the Nurses’ Health Study, a large prospective cohort study that consisted of US female nurses in their 50s at study enrollment, with repeated measures of service attendance and marital status over 14 years of follow-up from 1996–2010. During follow up, among 66,444 initially married nurses who were mainly Christians, frequent service attendance was associated with 50% lower risk of divorce (95% CI: 32%, 63%), and 52% lower risk of either divorce or separation (95%CI: 37%, 63%). Among initially divorced or separated women, frequent service attendance was not associated with subsequent likelihood of remarriage; however, among widowed women, women who attended services frequently had 49% increased likelihood of remarriage (95% CI: 13%, 97%) compared to those women who did not. The study provides evidence that in this cohort of US nurses, frequent service attendance is associated with lower risk of becoming divorced in mid- and late- life, and increased likelihood of remarriage among widowed nurses, but not among divorced or separated nurses.

## Introduction

The United States is a religious country. The Pew Research center reported that 92% U.S. adults in 2007 and 89% U.S. adults in 2012 believe in God, with a majority (71%) identifying as Christian [1]. Religion plays an important role in marriage and church attendance has been shown to be a strong predictor for remaining married versus divorcing [2]. Marriage, either marriage cohesiveness or divorce, are a special case of social groups cohesiveness [2], conferring psychological and economic benefits to couples and society [3–5]. However, despite these positive perspectives on marriage, the crude marriage rate declined from 8.2 per 1,000 total population in 2000 to 6.9 in 2015 [6]. Divorce rates have fallen somewhat over this time, with 4.0 per 1,000 reported in 2000 which declined to 3.1 in 2015 [6, 7]. Divorce rates in middle aged groups however doubled from 1990 to 2010 [8, 9]. There is some evidence that the tendency for divorced men and women, particularly aged 50 years older, to get remarried has increased in recent years [7, 10, 11]. Prior research has mainly focused on early-life divorces [7, 9, 12–14]. There is limited evidence on subsequent re-marriage among the increasingly growing group of late life divorcees. The baby boomers in the U.S. are now in mid- to late- life. With late-life divorce rate doubling in recent years, there is a need to better understand divorce and remarriage for mid- and late- life women [13, 15].

Classic Durkheimian theory suggests that the institutions of religion contribute to human well-being, fostering and supporting marriage; and married couples who are religious may be happier and more satisfied with life [7–11, 16–23]. Marriage may be viewed as an end in itself, but is also associated longitudinally with better physical and mental health and greater life satisfaction [4, 5, 24–34]. Although marital conflicts can sometimes seem irreconcilable, interventions, counseling, or shared activity that might be available earlier in the relationship, when problems first begin to develop, could be effective ways to prevent divorce and promote well-being [12, 35]. Current evidence on service attendance and the likelihood of remarriage after widowhood or separation is sparse. It is unclear whether religious service attendance is associated with subsequent likelihood of remarriage generally, and whether this depends on whether the marriage ended or someone was widowed.

In addition, other research has suggested religious participation can play an important role in promoting strong marital bonds, happiness, and stability [35–40]. On the other hand, religious attendance and affiliation may operate as a barrier to divorce [41, 42]. Religious service attendance might affect marital stability because of religious teaching of marriage as sacred, teachings against divorce, a place to meet other couples and families, and programs, counseling and retreats that support marriage and family life. Religious affiliation may affect family life because of different teachings and practices concerning marriage, divorce, and remarriage [35, 38]. Literature on remarriage has suggested that there are significant denominational subcultural variations for remarriage among Americans [15]. For example, conservative Protestants are most likely to remarry and least likely to cohabit [15, 43]. Moreover, remarriage after a divorce may be viewed less favorably than remarriage after widowhood or separation by some religious denominations [15, 35, 38]. Despite many qualitative studies that have examined religious participation, affiliation and marriage, the quantitative assessment of the joint effects of religious service attendance and religious affiliation on marriage is currently unclear. Here we will examine differences comparing Catholic and Protestant denominations.

A number of methodological concerns limit research to date in the field of religion and marriage. Most existing studies on religion and marital status have used cross-sectional data [40, 44, 45], which may be subject to reverse causation issues with divorce itself affecting subsequent service attendance [46]. Data from prospective cohort studies on the interrelationship between religion and marital status over time are sparse [38, 39, 47]. Contemporary data with longer follow up are needed. Prior research has also included only a limited set of potential confounders when examining these associations, leaving open the strong possibility of unmeasured and residual confounding. For example, most covariates considered in prior studies focused on socio-economic related variables. Health and lifestyle related information, such as, parity (*number of times that the nurse has given birth*), depression, or good physical function, were not available. Various lifestyle factors may affect both service attendance and marital conflict and stability, thereby confounding the association [25, 48–52]. In addition, existing literature has not taken into account prior religious service attendance, to further rule out issues of reverse causation.

To address these gaps in knowledge, we conducted a more rigorous test of the hypothesis that frequent religious service attendance is associated with a lower likelihood of divorce, and increased likelihood of remarriage among nurses in mid- to late- life. Using data from a large prospective cohort study of 66,444 US female nurses who were mainly Christians, we tested the following two hypotheses: 1) frequent religious service attendance is associated with lower subsequent odds of divorce or separation; and 2) frequent religious service attendance is associated with higher likelihood of remarriage. We also evaluated these associations by Catholic versus Protestant affiliation to assess the joint effects of attendance and affiliation.

## Materials and methods

### The Nurses’ Health Study

Our study sample was from the Nurses’ Health Study which began in 1976 when 121,700 married female registered nurses aged 30 to 55 years, completed a mailed questionnaire about their medical history, dietary and lifestyle habits. They resided in 1 of 11 US states with the largest number of registrants (New York, California, Pennsylvania, Ohio, Massachusetts, New Jersey, Michigan, Texas, Florida, Connecticut, and Maryland). Among those invited, 71.2% participants returned the questionnaire. The overall NHS in 1976 was 98% white, with a relatively higher socioeconomic status. While this limits generalizability, the homogeneity does enhance the interval validity of the NHS, as does also the high quality data as well as high follow-up rates [53].

Follow-up questionnaires were sent every 2 years, with follow-up for more than 90% the cohort remaining complete. Data on service attendance was available starting in 1992, 1996 and every four years subsequently; for these analyses we thus restricted the study population to nurses who were currently married in 1996 and also reported their religious service attendance information in 1996 (n = 66,444). For analyses on remarriage, we restricted the study population to nurses who were divorced (n = 7,311), separated (n = 986), or widowed (n = 21,372) in 1996 and also reported their religious service attendance information in 1996. Follow up data for these women is available through 2010. The Nurses’ Health Study was approved by the Brigham and Women’s hospital and Harvard T. H. Chan School of Public Health Institutional Review Board of (Boston, MA).

### Ascertainment of religious service attendance and religious affiliation

Information on religious service attendance was collected from self-administered questionnaires in 1992 and 1996, and every four years subsequently. The study participants were asked, “How often do you go to religious meetings or services?” Response categories included “more than once a week, once a week, 1–3 times per month, less than once per month, never or almost never.” Previous literature suggested that even though religious service attendance tends to be over-reported, it is still a good indicator of communal religious practice [54]. Moreover, relative rank ordering across frequency may still be preserved.

Religious affiliation was self-reported, with a majority of the NHS participants being Protestant or Catholic. Due to small numbers in other religious group in our study sample, we restricted our analyses on religious affiliation to women who reported being either “Protestant” or “Catholic”.

Religious service attendance was modeled as four categories: “more than once a week, once a week, less than once per week, never or almost never.”

### Ascertainment of divorce or separation

The NHS participants self-reported their marital status every four years from 1992 to 2010. We defined a divorce as the study participant self-reporting any divorce event during the follow up period from 1996 to 2010 (ever been divorced, yes or no) and evaluated both the presence and timing of such [55, 56]. Divorce or separation was defined by self-report as either divorce or separation during 1996–2010 (either being divorced or separated, yes or no). During the follow-up period, we identified 685 total events for self-reported divorce, and 924 total events for self-reported divorce or separation.

### Ascertainment of remarriage

We defined a remarriage event as women who previously reported being divorced, separated, or widowed at baseline 1996, but self-reported being currently married in any of the follow-up questionnaires from 2000 until 2010. Widowhood information was obtained through women’s self-reported questionnaire. We categorized remarriage among four groups of women: 1) all previously married women who self-reported as unmarried for any reason (i.e., divorced or separated or widowed) in 1996; 2) only women who self-reported being divorced in 1996; 3) only women who self-reported being separated in 1996; and 4) only women who self-reported as widows in 1996.

### Covariates

Potential confounders for the association between religious service attendance and marital status (divorce, remarriage) were selected *a priori* based on the literature and subject knowledge [57, 58]. We included the socio-demographic characteristics of the study participants, including husband’s education, median family income, geographic region, and unemployment in the past two years. We also evaluated lifestyle and health characteristics that associated with service attendance and marriage, self-reported prior religious attendance frequency (never, < 1/week, > 1/week in 1992), and prior divorce history (yes, no). We included the following various health-related lifestyle factors and health conditions because health may affect both service attendance and marital conflict and stability: alcohol consumption and healthy eating index measured by a validated food frequency questionnaire, good physical or function measured by the 36-Item Short Form Health Survey (SF-36), depression measured by the Center for Epidemiologic Studies Depression -10 Scale, parity (*number of times that the nurse has given birth*), physical exercise, hypertension, hypercholesterolemia, type 2 diabetes, menopausal status and postmenopausal hormone use, physical exam in the past 2 years, smoking status, pack-years, and body mass index (BMI). Prior history of divorce was defined as “ever reported divorce in the past 20 years (1976–1996 (our analytic baseline)).

### Statistical analyses

The primary exposure was religious service attendance in 1996, with prior attendance in 1992 controlled for as a covariate in an effort to mitigate concerns about reverse causation. Cox proportional hazard model and multivariate logistic regression was used to estimate the longitudinal association between the 1996 service attendance and likelihood of divorce/separation over the follow-up period. Model 1 was age adjusted. Model 2, the fully adjusted model, included baseline religious service attendance level in 1992, demographics (age, husband’s education, median family income, geographic region, unemployed in past two years, prior history of divorce), health conditions (parity (*Number of times that nurses has given birth to a fetus with a gestational age of 24 weeks or more*), hypertension, hypercholesterolemia, type 2 diabetes, menopausal status, postmenopausal hormone use, physical function, depression status), health lifestyle behaviors (smoking status, pack years, physical exercise, alcohol consumption, healthy diet, physical exam in past 2 years, BMI). For Cox model, we also adjusted for calendar year and questionnaire cycle. We tested proportional hazard assumption by adding an interaction term between services attendance and time.

We performed interaction subgroup analyses comparing self-identified Protestants and Catholics. Specifically, we evaluated interaction both on multiplicative and additive scales (using the relative excess risk due to interaction) [59, 60]. As a sensitivity analyses, we further used propensity score subclassification by quintiles to obtain effect estimates (comparing ever vs. never service attendance). We also assessed how substantial unmeasured confounding (for example, unmeasured marital status of the nurses’ parents in childhood) would have to be to explain away the associations [46].

## Results and discussion

### Association of religious service attendance with subsequent divorce

Of 66,444 women who were currently married at baseline year 1996, 924 of them became either divorced (n = 685), or separated (n = 239) from 1996–2010. Compared to women who never attended religious services, those who attended more than once per week in 1996 had lower prevalence of depression, drank less alcohol in midlife, were less likely to be current smoker, and less likely to be nulliparous (Table 1). The majority of our study participants were Caucasian (98%, S1 Table).

**Table 1:** Age-adjusted baseline characteristics of study participants by frequency of religious service attendance in 1996.

	Religious service attendance in 1996			
	Almost never (n = 15334)	Less than once/week (n = 10557)	Once/week (n = 27951)	More than once/week (n = 12602)
Age at 1996,year*	52.76 (7.00)	52.32 (7.09)	53.45 (6.95)	54.40 (6.82)
Caucasians, %	98	98	98	98
Religious group				
Catholic, %	29	27	54	40
Protestant, %	63	63	43	53
Other Christian, %	2	2	2	6
Ashkenazi Jewish, %	4	7	1	0
Sephardic Jewish, %	0	0	0	0
Eastern (e.g. Buddhist, Hindu), %	0	0	0	0
Muslim, %	0	0	0	0
Other religious heritage, %	2	1	1	1
Husband’s education level				
Less than high school	1	1	1	2
Some high school	3	3	3	3
High school graduate	32	31	33	33
Graduate school	22	23	20	22
Missing	16	17	18	17
Not employed in last 2 years, %	42	39	40	44
Baseline depression, %	7	6	5	3
Geographic region, %				
North, %	37	37	36	33
South, %	13	10	9	13
Middle, %	41	43	45	44
Body mass index, kg/m^2^	26.56 (5.50)	26.67 (5.22)	26.49 (5.07)	26.52 (5.11)
Physical activity, MET-hrs/wk	17.56(23.05)	18.02(21.69)	17.37(21.69)	17.46(20.27)
Smoking status, %				
Past smoker < 10 pack years	16	17	17	16
Past smoker 10–19 pack years	10	10	10	8
Past smoker 20–39 pack years	13	11	10	8
Past smoker 40+ pack years	8	6	5	3
Past smoker unknown pack years	1	1	1	1
Current smoker < 25 pack years	2	2	2	1
Current smoker 25–44 pack years	6	5	3	2
Current smoker 45–64 pack years	5	3	3	1
Current smoker 65+ pack years	3	1	1	0
Diabetes, %	17	16	15	15
Hypertension, %	42	42	40	39
Hypercholesterolemia, %	54	55	55	54
Post-menopausal hormone use, %				
Never user	19	18	20	19
Current user	16	16	14	16
Past user	50	52	52	53
No physical function limitation, %	48	49	51	50
Alcohol consumption, g/day				
0.1–4.9 g/d, %	27	30	30	26
5.0–14.9 g/d, %	19	19	17	13
15.0+ g/d, %	12	9	7	5
Nulliparous, %	7	4	5	5
Prior history of divorce, %	1	1	1	0
Median family income, dollars/year	68266(27621)	67898(27649)	63508(24108)	61728(24118)

Values are means(SD) or percentages and are standardized to the age distribution of the study population.

* Value is not age adjusted.

Compared to women who never attended religious services, those who attended services more than once per week were 42% less likely to get divorced, or 47% less likely to get either divorced or separated (HR = 0.58, 95% CI: 0.44–0.74, p trend <0.0001 for divorce only, HR = 0.53, 95% CI: 0.42–0.67, p trend <0.0001 for divorce or separation, Table 2). Results were similar using propensity score stratification by quintiles (HR = 0.53, 95% CI: 0.41–0.69 for divorce only, HR = 0.49, 95% CI: 0.39–0.62 for divorce or separation). We also found similar results when using logistic regression mode (OR = 0.50, 95% CI: 0.37–0.68, p trend <0.0001 for divorce only, OR = 0.48, 95% CI: 0.37–0.63, p trend <0.0001 for divorce or separation, S2 Table).

**Table 2:** Multivariate adjusted association between religious services attendance and subsequent divorce or separation in the Nurses’ Health Study, 1996–2010.

	Religious service attendance in 1996				P trend
	Never	Less than once/week	Once/week	More than once/week	
Outcome: divorce only					
Divorce cases No. = 685	236	152	218	79	
Age-adjusted HR (95% CI)	1.00 (ref)	0.88 (0.72, 1.08)	0.54 (0.45, 0.65)	0.49 (0.38, 0.63)	<0.0001
Multivariable HR (95% CI)*	1.00 (ref)	0.93 (0.76, 1.15)	0.61 (0.50, 0.74)	0.58 (0.44, 0.74)	<0.0001
Outcome: divorce or separation					
Divorce or separation cases No. = 924	320	201	297	106	
Age-adjusted HR (95% CI)	1.00 (ref)	0.86 (0.72, 1.03)	0.54 (0.46, 0.64)	0.47 (0.38, 0.59)	<0.0001
Multivariable HR (95% CI)*	1.00 (ref)	0.90 (0.75, 1.07)	0.59 (0.50, 0.69)	0.53 (0.42, 0.67)	<0.0001

CI: confidence interval

HR: hazard ratio

* Multivariable model adjusted for age (continuous), calendar year, questionnaire cycle, alcohol consumption (none, 0.1–4.9, 5.0–14.9, ≥15.0 g/d), husband’s education (less than high school, some high school, high school graduate, college, graduate school), good physical or function (yes, no), median family income(dollars/year), geographic region (north, south, middle, other) and religious service attendance in 1992 (never, < 1/week, > 1/week), unemployed in the past two years (yes, no), baseline depression (yes, no), parity (nulliparous, 1–2, 3–4, 5+), prior history of divorce (yes, no), physical exercise (metabolic equivalent values; quintiles), hypertension (yes, no), hypercholesterolemia (yes, no), type 2 diabetes (yes, no), menopausal status (premenopausal, postmenopausal) and postmenopausal hormone use (never, past and current), physical exam in the past 2 years (no, yes for symptoms and yes for screenings), healthy eating score (quintiles), smoking status (never, former, current), pack-years (<10, 10–19, 20–39, ≥40 for former smokers; <25, 25–44, 45–64, ≥65 for current smokers), and BMI (kg/m^2^; <21, 21–22.9, 23–24.9, 25–27.4, 27.5–29.9, 30–34.9, ≥35).

In our study, we did not have information on duration of current marriage, which may be a potential unmeasured confounder. This is so because there is some evidence that religious people tend to marry young, and divorce rate varies by marriage duration. However, for unmeasured confounding to explain away the odds ratio estimate of 0.50 for divorce, an unmeasured confounder associated with both divorce and regular religious service attendance by risk ratios of 3.4-fold each, above and beyond the measured confounders, could do so but weaker confounding could not. For an unmeasured confounder to bring the upper confidence limit of 0.68 for this estimate above 1, an unmeasured confounder that was associated with both divorce and regular service attendance by risk ratios of 2.3-fold each could do so, but weaker confounding could not. Similar substantial confounding would be needed to explain away the other estimates.

We further examined the joint effects of service attendance and religious affiliation. We found multiplicative interaction for divorce. Attending religious services once or more per week was associated with 52% lower (95% CI: 40%-66%) likelihood of divorce for Catholics, but only a 32% lower (95% CI: 10%-43%) likelihood of divorce for Protestants (p-value for multiplicative interaction = 0.02). The large effect size for Catholics was in part due to the higher likelihood of divorce for those not attending (Table 3, S3 Table).

**Table 3:** Joint effect of religious service attendance in 1996 and religious affiliation and subsequent divorce or separate.

Frequency of religious service attendance	Religious affiliation		HRs (95% CI) for religious affiliation within strata of religious service attendance*
	Protestant	Catholic	
	HR (95% CI)	HR (95% CI)	
Subsequent divorce			
Never or < once/week	1.0	1.15 (0.92–1.43)	1.15 (0.92, 1.43)
≥ once/week	0.72 (0.57–0.90)	0.59 (0.47–0.74)	0.83 (0.64–1.06)
HRs (95%CI) for service attendance within strata of religious affiliation*	0.72 (0.57–0.90)	0.52 (0.40–0.66)	
Measure of effect modification on additive scale: RERI (95%CI) = -0.35 (-0.75, 0.04); p = 0.08. The multiplicative interaction and its 95% CI = 0.66 (0.45, 0.96). P value for multiplicative interaction = 0.02			
Subsequent divorce or separation			
Never or < once/week	1.0	1.17 (0.97–1.42)	1.17 (0.97–1.42)
≥ once/week	0.67 (0.55–0.82)	0.62 (0.51–0.75)	0.92 (0.74–1.14)
HRs (95%CI) for service attendance within strata of religious affiliation*	0.67 (0.55–0.82)	0.53 (0.43–0.65)	
Measure of effect modification on additive scale: RERI (95%CI) = -0.24 (-0.56, 0.08); p = 0.14. The multiplicative interaction and its 95% CI = 0.76 (0.55, 1.05). P value for multiplicative interaction = 0.09			

CI: confidence interval

HR: hazard ratio

* Multivariable model adjusted for age (continuous), calendar year, questionnaire cycle, alcohol consumption (none, 0.1–4.9, 5.0–14.9, ≥15.0 g/d), husband’s education (less than high school, some high school, high school graduate, college, graduate school), good physical or function (yes, no), median family income(dollars/year), geographic region (north, south, middle, other) and religious service attendance in 1992 (never, < 1/week, > 1/week), unemployed in the past two years (yes, no), baseline depression (yes, no), parity (nulliparous, 1–2, 3–4, 5+), prior history of divorce (yes, no), physical exercise (metabolic equivalent values; quintiles), hypertension (yes, no), hypercholesterolemia (yes, no), type 2 diabetes (yes, no), menopausal status (premenopausal, postmenopausal) and postmenopausal hormone use (never, past and current), physical exam in the past 2 years (no, yes for symptoms and yes for screenings), healthy eating score (quintiles), smoking status (never, former, current), pack-years (<10, 10–19, 20–39, ≥40 for former smokers; <25, 25–44, 45–64, ≥65 for current smokers), and BMI (kg/m^2^; <21, 21–22.9, 23–24.9, 25–27.4, 27.5–29.9, 30–34.9, ≥35).

### Association of religious service attendance with subsequent remarriage

During the follow-up from 1996–2010, we identified 1567 remarriage events among women who had divorced, separated, or widowed in 1996, with a total of 723 events among women divorced in 1996, 197 events among women separated in 1996, and 695 events among women widowed in 1996.

Women who attended religious services once per week or more, and who were widows in 1996, had significantly higher likelihood of remarriage (HR = 2.06, 95% CI: 1.52–2.80, *p for trend* ≤ 0.001, Table 4), but those who were divorced or separated did not. Results were similar using logistic regression model (S4 Table) or propensity score subclassification by quintiles (OR = 1.50, 95% CI: 1.17–1.93 for remarriage after widowhood; OR = 0.92, 95% CI: 0.70–1.20 for remarriage after divorce, OR = 0.74, 95% CI: 0.44–1.25 for remarriage after separation). In examining the joint effects of religious service attendance and religious affiliation on remarriage, there was perhaps some indication that Catholics regularly attending religious services were less likely to remarry than Protestants when results were aggregated over divorced, widowed, and separated women (Table 5, S5 Table). Among women who attended services once or more per week, Catholics had 34% lower likelihood of remarriage than Protestants (95% CI: 22%-45%; p value for multiplicative interaction = 0.09, p value for additive interaction = 0.06).

**Table 4:** Multivariate adjusted association between religious services attendance and subsequent remarriage in the Nurses’ Health Study, 1996–2010.

	Religious service attendance in 1996				P trend
	Never	Less than once/week	Once/week	More than once/week	
Among widowed women in 1996					
Remarriage cases No. = 695	116	118	294	167	
Age-adjusted HR (95% CI)	1.00 (ref)	1.74 (1.25–2.43)	2.36 (1.78–3.11)	3.15 (2.34–4.26)	<0.0001
Multivariable HR (95% CI)*	1.00 (ref)	1.48 (1.06–2.06)	1.69 (1.27–2.24)	2.06 (1.52–2.80)	<0.001
Among divorced women in 1996					
Remarriage cases No. = 723	247	184	215	77	
Age-adjusted HR (95% CI)	1.00 (ref)	1.22 (1.00–1.49)	0.94 (0.78–1.14)	0.85 (0.65–1.11)	0.16
Multivariable HR (95% CI)*	1.00 (ref)	1.23 (1.00–1.50)	1.02 (0.84–1.24)	0.91 (0.70–1.19)	0.60
Among women who had separation in 1996					
Remarriage cases No. = 197	70	44	58	25	
Age-adjusted HR (95% CI)	1.00 (ref)	0.98 (0.61–1.57)	0.86 (0.56–1.31)	1.00 (0.59–1.69)	0.71
Multivariable HR (95% CI)*	1.00 (ref)	0.90 (0.56–1.44)	0.66 (0.43–1.02)	0.72 (0.42–1.23)	0.07
Among women who had previously married and self-reported as unmarried in 1996					
Remarriage cases No. = 1567	423	334	551	259	
Age-adjusted HR (95% CI)	1.00 (ref)	1.31 (1.09–1.56)	1.24(1.06–1.45)	1.39 (1.15–1.69)	0.0009
Multivariable HR (95% CI)*	1.00 (ref)	1.24 (1.04–1.48)	1.14 (0.97–1.34)	1.21 (0.99–1.47)	0.08

CI: confidence interval

HR: hazard ratio

* Multivariable model adjusted for age (continuous), calendar year, questionnaire cycle, alcohol consumption (none, 0.1–4.9, 5.0–14.9, ≥15.0 g/d), husband’s education (less than high school, some high school, high school graduate, college, graduate school), good physical or function (yes, no), median family income(dollars/year), geographic region (north, south, middle, other) and religious service attendance in 1992 (never, < 1/week, > 1/week), unemployed in the past two years (yes, no), baseline depression (yes, no), parity (nulliparous, 1–2, 3–4, 5+), prior history of divorce (yes, no), physical exercise (metabolic equivalent values; quintiles), hypertension (yes, no), hypercholesterolemia (yes, no), type 2 diabetes (yes, no), menopausal status (premenopausal, postmenopausal) and postmenopausal hormone use (never, past and current), physical exam in the past 2 years (no, yes for symptoms and yes for screenings), healthy eating score (quintiles), smoking status (never, former, current), pack-years (<10, 10–19, 20–39, ≥40 for former smokers; <25, 25–44, 45–64, ≥65 for current smokers), and BMI (kg/m^2^; <21, 21–22.9, 23–24.9, 25–27.4, 27.5–29.9, 30–34.9, ≥35).

**Table 5:** Joint effect of religious service attendance in 1996 and religious affiliation on subsequent remarriage.

Frequency of religious service attendance	Religious affiliation		HRs (95% CI) for religious affiliation within strata of religious service attendance*
	Protestant	Catholic	
	HR (95% CI)	HR (95% CI)	
Remarriage among widowed women in 1996			
Never or < once/week	1.0	0.81 (0.54–1.20)	0.81 (0.54–1.20)
≥ once/week	1.73 (1.32–2.26)	1.14 (0.86–1.52)	0.66 (0.52–0.84)
HRs (95%CI) for service attendance within strata of religious affiliation*	1.73 (1.32–2.26)	1.42 (0.97–2.07)	
Measure of effect modification on additive scale: RERI (95%CI) = -0.37 (-1.05, 0.30); p = 0.28. The multiplicative interaction and its 95% CI = 0.92 (0.49, 1.72). P value for multiplicative interaction = 0.79			
Remarriage among divorced women in 1996			
Never or < once/week	1.0	0.98 (0.80–1.21)	0.98 (0.80–1.21)
≥ once/week	1.15 (0.93–1.41)	0.74 (0.59–0.93)	0.72 (0.57–0.91)
HRs (95%CI) for service attendance within strata of religious affiliation*	1.15 (0.93–1.41)	0.78 (0.62–0.99)	
Measure of effect modification on additive scale: RERI (95%CI) = -0.37 (-0.78, 0.05); p = 0.08. The multiplicative interaction and its 95% CI = 0.69 (0.47, 1.02). P value for multiplicative interaction = 0.06			
Remarriage among women who were separated in 1996			
Never or < once/week	1.0	0.77 (0.46–1.29)	0.77 (0.46–1.29)
≥ once/week	0.66 (0.40–1.09)	0.74 (0.47–1.15)	1.08 (0.67–1.76)
HRs (95%CI) for service attendance within strata of religious affiliation*	0.66 (0.40–1.09)	1.00 (0.61–1.64)	
Measure of effect modification on additive scale: RERI (95%CI) = 0.41 (-0.15, 0.98); p = 0.15. The multiplicative interaction and its 95% CI = 1.72 (0.71, 4.15). P value for multiplicative interaction = 0.23			
Remarriage among women who had previously married and self-reported as unmarried in 1996			
Never or < once/week	1.0	0.88 (0.73–1.06)	0.88 (0.73–1.06)
≥ once/week	1.24 (1.05–1.45)	0.82 (0.69–0.97)	0.66 (0.55–0.78)
HRs (95%CI) for service attendance within strata of religious affiliation*	1.24 (1.05–1.45)	0.93 (0.76–1.13)	
Measure of effect modification on additive scale: RERI (95%CI) = -0.29 (-0.62, 0.02); p = 0.06. The multiplicative interaction and its 95% CI = 0.78 (0.58, 1.04). P value for multiplicative interaction = 0.09			

CI: confidence interval

HR: hazard ratio

* Multivariable model adjusted for age (continuous), calendar year, questionnaire cycle, alcohol consumption (none, 0.1–4.9, 5.0–14.9, ≥15.0 g/d), husband’s education (less than high school, some high school, high school graduate, college, graduate school), good physical or function (yes, no), median family income(dollars/year), geographic region (north, south, middle, other) and religious service attendance in 1992 (never, < 1/week, > 1/week), unemployed in the past two years (yes, no), baseline depression (yes, no), parity (nulliparous, 1–2, 3–4, 5+), prior history of divorce (yes, no), physical exercise (metabolic equivalent values; quintiles), hypertension (yes, no), hypercholesterolemia (yes, no), type 2 diabetes (yes, no), menopausal status (premenopausal, postmenopausal) and postmenopausal hormone use (never, past and current), physical exam in the past 2 years (no, yes for symptoms and yes for screenings), healthy eating score (quintiles), smoking status (never, former, current), pack-years (<10, 10–19, 20–39, ≥40 for former smokers; <25, 25–44, 45–64, ≥65 for current smokers), and BMI (kg/m^2^; <21, 21–22.9, 23–24.9, 25–27.4, 27.5–29.9, 30–34.9, ≥35).

## Discussion

In this large prospective cohort study of 66,444 married U.S. nurses with 14 years of follow up from mid to late life, and repeated measurements of service attendance to allow for control of prior service attendance, nurses who attended religious service more than once per week had a 50% lower likelihood of subsequent divorce or separation, compared to those who never attend. Among widowed nurses, those who attended services more than once per week had a 49% higher likelihood of remarriage, compared to those who never attended services. However, for divorced or separated -nurses, religious service attendance was not significantly associated with the likelihood of remarriage. We found the associations between religious service attendance divorce, and remarriage were stronger for Catholics than Protestants.

Existing research on religion and marriage has mainly focused on early-life divorces [7, 9, 12–14]. Currently little is known regarding late life marriage, divorce and remarriage. Given the increasingly growing group of late life divorcees and with the late-life divorce rate doubling in recent years, there is a need to advance our understanding regarding divorce and remarriage for mid- and late- life women [13, 15]. Social science literature on religiosity and marital status has been based mostly on two measures of religious participation: service attendance [16, 47, 61–64] and religious affiliation [16, 57, 58, 65]. Religious service attendance might affect divorce and remarriage because of religious teaching of marriage as sacred, marriage counseling, and providing a place to meet other couples and families, and support family life. There is evidence that religiosity reduces the likelihood of marital infidelity among these couples, and may further indirectly reduce the likelihood of subsequent divorce by increasing levels of marital satisfaction [36]. Religious service attendance had previously been shown to be predictive of stronger marital bonds, greater marital happiness, and stability [38, 66]. In our study, we found that among a group of US nurses in their 50s, those who attended religious services more than once per week had a 50% lower likelihood of subsequent divorce or separation, compared to those who never attend. Our results on associations between service attendance and divorce are consistent with findings from previous research [7, 38, 67]. We expanded prior literature on this by focusing on mid and late life marriage and divorce.

Evidence on religious participation and late life divorce is sparse, but the literature on studies of remarriage after divorce or widowhood are even more limited [15, 68–70]. Remarriage is a complex and understudied process [71, 72]. Bohannan proposed six important developmental tasks of remarriage including emotional, psychic, community, parental, economic, and legal aspects of remarriage [71]. Compared with first marriages, remarriage has its own distinct assortative mating patterns and gender specific dynamics [73, 74]. For a first marriage, both men and women emphasize spousal economic status as a valuable trait; however, for remarriage, high-status men tend to marry less-educated, young women regardless of economic status [73].

To the best of our knowledge, there has been no prior study focused on the link between religion and remarriage in mid- and late- life, a period in which most divorces/remarriage occurs. Only one study has prospectively examined the effects of religion on remarriage, but was focused on young adulthood [15]. The authors reported a positive association between religious service attendance and likelihood of remarriage among American women aged 15–44 years, and reported differences in religious affiliations [15]. However, this study was based on pooling of two cross-sectional surveys in 1988 and 1994. It is unknown whether this observation is still true with longitudinal data, longer follow-up, and among contemporary elderly Americans. Our study provided evidence by using a prospective cohort design with contemporary data 1996–2010, repeated measurements of service attendance and marital status, and controlling for past service attendance in an effort to rule out possible reverse causation [46, 75]. In our study, we were able to examine these associations among US nurses in their 50’s, an age group of women that has previously been understudied. We found that among widowed nurses, those who attended services more than once per week had a 49% higher likelihood of remarriage, compared to those who never attended services. However, the associations were not significant for those women who were divorced or separated. Religion may place a certain stigma on divorce [67]. Catholic teaching prohibits remarriage after divorce, unless an annulment of the prior marriage takes place. Such teachings may in part contribute to the lower rate of divorce, but may also help explain the result that service attendance is only associated with high likelihood of remarriage for those who are widowed but not for those who are divorced or separated [76]. Another possible explanation is that women who experience a divorce or separation event may have been extremely unhappy in marriage, and thus less likely to enter a marriage again. In contrast, a woman who is a widow may benefit from the support from religious community, and may be more willing to remarry.

Strengths of our study include large sample size, prospective cohort design, long duration of follow-up, availability of repeated measures of marital status and religious service attendance, extensive covariates control, with over a 90% follow-up rate. We provided long-term evidence on religion, divorce, and remarriage among contemporary Americans. In our study, we were able to examine these associations among US nurses in their 50’s, an age group of women that has previously been understudied. Our study sample included only U.S. nurses and consisted mostly of Caucasian healthcare professionals. Compared with the general population, they generally have higher levels of education and socioeconomic status. Our results may not therefore be generalizable to the overall general population in the US, and women in other race/ethnicity, age, and cultural groups. However, this relative homogenous group does ensure excellent internal validity of our study. Further, our sensitivity analysis suggested that the results were robust to potential unmeasured confounding, such as marital duration. Although our study was an observational study, we adjusted for a number of major potential confounders of the association between religious service attendance and divorce. The results from sensitivity analyses were relatively robust. Another strength of our study is that we have detailed lifestyle factor information and were able to additionally account for the confounding factors by these variables, such as depression, and good physical function, as literature has suggested that marriage promotes health and healthy behavior which may in turn affect marital stability.

Existing literature suggests the following variables are relevant predictors for divorce: marrying as a teenager, poverty, unemployment, low education, living with one’s future spouse or another partner prior to marriage, having a premarital birth, bringing children from a previous union into a new marriage, inter-racial marriage, and growing up in a household without two continuously married parents [2, 7, 77]. We did not have data on all of these and some of these might also be related to religious service attendance. Introducing unmeasured confounding For example, religious participation is also associated with earlier timing of marriage [78]: those who attend religious services more frequently and those who report religion being important are more likely to get married at a younger age [79], which is itself a risk factor for divorce. In our study, we did not have detailed information about the duration of a study participant’s current marriage, and age at first marriage. However, we did perform sensitivity analyses to assess how strong unmeasured confounding would have to be to explain away the observed association. For an unmeasured confounder to explain away the association of service attendance with divorce, an unmeasured confounder would need to be associated with both divorce and regular religious service attendance by risk ratios of 3.4-fold each, above and beyond the measured covariates. Such substantial confounding by unmeasured factors seems very unlikely given adjustment for an extensive set of covariates and relatively homogenous study population in the NHS study.

Our study is subject to certain other limitations as well. First, men and women perceive marriage differently. Previous studies have suggested that there is a gender difference in perceptions of martial problems [7] and attitudes toward remarriage. The association we observed between religiosity and divorce/remarriage might be gender-specific. Our study focused on wives’ religious participation only. Currently, very few studies have information regarding the religiosity of both spouses, and joint service attendance [38, 80]. Future studies on religious participation and divorce/remarriage specifically for men, and for men and women jointly are needed. Second, service attendance in our study was self-reported but this has been widely used in the sociology literature and may at least preserve relative ordering of attendance. In our study, marital status was measured through self-reported questionnaire. For women who experienced both divorce and remarriage events within a 4-year interval, we were not be able to capture these events and may have misclassified them as remaining married. However, such misclassifications are probably rare. In the NHS, information was not collected regarding whether the couples were of the same sex. At the time of the NHS study baseline, same-sex couple were likely not common among married women and such misclassification would likely be rare. Third, religiosity is a multi-dimensional construct. One single measure of service attendance does not capture other aspects of religiosity, such as beliefs in religious teaching, personal spirituality, and practices of prayer or joint prayer with one’s spouse. In our study, religious affiliation information was collected through self-report and we could not separate out Protestants into conservative and mainline Protestants, as more specific groups may have heterogeneous effects. Further, the majority of our study participants reported being “Protestant” or “Catholic”, and very few women reported being in other religious groups. Our results may not generalizable to people with different cultural backgrounds, and religious beliefs. Fourth, some have argued that even though religiosity and religious service attendance may increase marital quality, and decrease marital problems [81], it may also increase the stigma associated with divorce [7] and perhaps thereby further decreases the likelihood of divorce [82]. In our study among US female nurses, we did not have marital quality and satisfaction information and it is possible that some women remained married despite low satisfaction with their marriage [83]. Future studies are needed to examine the association between frequency of religious service attendance, divorce stigma, and marital quality and satisfaction. Future studies on both spouses are still needed.

## Conclusions

In conclusion, in this prospective cohort study of U.S. female nurses over a 14-year period of follow-up from mid- to late- life, we found that frequent religious services attendance halved the risk of divorce among married women. Additionally, frequent religious service attendance was also associated with increased likelihood of remarriage among widowed women, but not for divorced or separated women. Future studies on religion and divorce/remarriage are needed with other religious beliefs other than Christianity, with other dimensions of religiosity, information on both couples, and the incorporation of information on marital satisfaction and quality.

## Supporting information

S1 Table Characteristics of the Nurses’ Health Study participants included and excluded in the analytic sample. (DOCX)

S2 Table Multivariate adjusted association between religious services attendance and subsequent divorce or separation in the Nurses’ Health Study, 1996–2010. (DOCX)

S3 Table Joint effect of religious service attendance in 1996 and religious affiliation and subsequent divorce or separate. (DOCX)

S4 Table Multivariate adjusted association between religious services attendance and subsequent remarriage in the Nurses’ Health Study, 1996–2010. (DOCX)

S5 Table Joint effect of religious service attendance in 1996 and religious affiliation on subsequent remarriage. (DOCX)
