# Supplementary material for: Religious service attendance, divorce, and remarriage among U.S. nurses in mid and late life
Source: PLoS One. 2018 Dec 3;13(12):e0207778. doi: 10.1371/journal.pone.0207778 (PMC6277070; doi:10.1371/journal.pone.0207778)
Supplement: S1 Table — (DOCX) [file pone.0207778.s001.docx]

**Supporting information**

S1 Table. Characteristics of the Nurses’ Health Study participants included and excluded in the analytic sample

Results from logistic regression model:

S2 Table. Multivariate adjusted association between religious services attendance and subsequent divorce or separation in the Nurses’ Health Study, 1996-2010

S3 Table. Joint effect of religious service attendance in 1996 and religious affiliation and subsequent divorce or separate

S4 Table. Multivariate adjusted association between religious services attendance and subsequent remarriage in the Nurses’ Health Study, 1996-2010

S5 Table. Joint effect of religious service attendance in 1996 and religious affiliation on subsequent remarriage

S1 Table. Characteristics of the Nurses’ Health Study participants included and excluded in the analytic sample

|  | Excluded from the analytic sample | Included in the analytic sample |
| --- | --- | --- |
| Age at 1996, year^*^ | 62.26(7.12) | 62.60(7.22) |
| Caucasians, % | 98 | 95 |
| Religious group |  |  |
| Catholic, % | 42 | 48 |
| Protestant, % | 52 | 46 |
| Other Christian, % | 3 | 2 |
| Ashkenazi Jewish, % | 2 | 2 |
| Sephardic Jewish, % | 0 | 0 |
| Eastern (e.g. Buddhist, Hindu), % | 0 | 1 |
| Muslim, % | 0 | 0 |
| Other religious heritage, % | 1 | 2 |
| Husband’s education level |  |  |
| Less than high school | 2 | 1 |
| Some high school | 3 | 2 |
| High school graduate | 31 | 15 |
| Graduate school | 19 | 9 |
| Missing | 22 | 63 |
| Not employed in last 2 years, % | 41 | 33 |
| Baseline depression, % | 6 | 3 |
| Geographic region, % |  |  |
| North, % | 36 | 18 |
| South, % | 11 | 6 |
| Middle, % | 43 | 22 |
| Body mass index, kg/m² | 26.54(5.27) | 27.10(5.54) |
| Physical activity, MET-hrs/wk | 17.46(21.79) | 19.97(27.66) |
| Smoking status, % |  |  |
| Past smoker < 10 pack years | 16 | 15 |
| Past smoker 10-19 pack years | 9 | 8 |
| Past smoker 20-39 pack years | 11 | 10 |
| Past smoker 40+ pack years | 6 | 6 |
| Past smoker unknown pack years | 1 | 2 |
| Current smoker < 25 pack years | 2 | 2 |
| Current smoker 25-44 pack years | 5 | 4 |
| Current smoker 45-64 pack years | 3 | 4 |
| Current smoker 65+ pack years | 2 | 3 |
| Hypertension, % | 42 | 46 |
| Hypercholesterolemia, % | 55 | 52 |
| Post-menopausal hormone use, % |  |  |
| Never user | 25 | 24 |
| Current user | 39 | 34 |
| Past user | 20 | 16 |
| No physical function limitation, % | 48 | 37 |
| Multiple vitamin use, % | 53 | 2 |
| Alcohol consumption, g/day |  |  |
| 0.1-4.9 g/d, % | 29 | 12 |
| 5.0-14.9 g/d, % | 17 | 6 |
| 15.0+ g/d, % | 8 | 3 |
| Prior history of divorce, % | 6 | 4 |
| Median family income, dollars/year | 64406(25375) | 63771(25843) |

* Value is not age adjusted
