# Supplementary material for: Religious service attendance, divorce, and remarriage among U.S. nurses in mid and late life
Source: PLoS One. 2018 Dec 3;13(12):e0207778. doi: 10.1371/journal.pone.0207778 (PMC6277070; doi:10.1371/journal.pone.0207778)
Supplement: S2 Table — (DOCX) [file pone.0207778.s002.docx]

S2 Table. Multivariate adjusted association between religious services attendance and subsequent divorce or separation in the Nurses’ Health Study, 1996-2010

|  | Religious service attendance in 1996 | | | |  |
| --- | --- | --- | --- | --- | --- |
|  | Never | Less than once/week | Once/week | More than once/week | P trend |
| Outcome: divorce only |  |  |  |  |  |
| Divorce cases No. =685 | 236 | 152 | 218 | 79 |  |
| Age-adjusted OR (95% CI) | 1.00 (ref) | 0.94 (0.76-1.15) | 0.50 (0.42-0.61) | 0.40 (0.31-0.52) | <0.0001 |
| Multivariable OR (95% CI)^*^ | 1.00 (ref) | 0.98 (0.78-1.23) | 0.57 (0.46-0.71) | 0.50 (0.37-0.68) | <0.0001 |
|  |  |  |  |  |  |
| Outcome: divorce or separation |  |  |  |  |  |
| Divorce or separation cases No. =924 | 320 | 201 | 297 | 106 |  |
| Age-adjusted OR (95% CI) | 1.00 (ref) | 0.91 (0.76-1.09) | 0.50 (0.43-0.59) | 0.39 (0.32-0.50) | <0.0001 |
| Multivariable OR (95% CI)^*^ | 1.00 (ref) | 0.94 (0.77-1.15) | 0.56 (0.47-0.68) | 0.48 (0.37-0.63) | <0.0001 |

CI: confidence interval

OR: odds ratio

* Multivariable logistic regression model adjusted for age (continuous), calendar year, questionnaire cycle, alcohol consumption (none, 0.1-4.9, 5.0-14.9, ≥15.0 g/d), husband’s education (less than high school, some high school, high school graduate, college, graduate school), good physical or function (yes, no), median family income(dollars/year), geographic region (north, south, middle, other) and religious service attendance in 1992 (never, < 1/week, > 1/week), unemployed in the past two years (yes, no), baseline depression (yes, no), parity (nulliparous, 1-2, 3-4, 5+), prior history of divorce (yes, no), physical exercise (metabolic equivalent values; quintiles), hypertension (yes, no), hypercholesterolemia (yes, no), type 2 diabetes (yes, no), menopausal status (premenopausal, postmenopausal) and postmenopausal hormone use (never, past and current), physical exam in the past 2 years (no , yes for symptoms and yes for screenings), healthy eating score (quintiles), smoking status (never, former, current), pack-years (<10, 10-19, 20-39, ≥40 for former smokers; <25, 25-44, 45-64, ≥65 for current smokers), and BMI (kg/m^2^; <21, 21-22.9, 23-24.9, 25-27.4, 27.5-29.9, 30-34.9, ≥35).
