# Supplementary material for: Religious service attendance, divorce, and remarriage among U.S. nurses in mid and late life
Source: PLoS One. 2018 Dec 3;13(12):e0207778. doi: 10.1371/journal.pone.0207778 (PMC6277070; doi:10.1371/journal.pone.0207778)
Supplement: S3 Table — (DOCX) [file pone.0207778.s003.docx]

S3 Table. Joint effect of religious service attendance in 1996 and religious affiliation and subsequent divorce or separate

|  | Religious affiliation | | | |  |
| --- | --- | --- | --- | --- | --- |
|  | Protestant | | Catholic | | HRs (95% CI) for religious affiliation within strata of religious service attendance |
| Frequency of religious service attendance |  | OR (95% CI) |  | OR (95% CI) |  |
| Subsequent divorce | | | | | |
| Never or < once/week |  | 1.0 |  | 1.24 (0.98-1.58) | 1.24 (0.98-1.58) |
| > once/week |  | 0.67 (0.52-0.85) |  | 0.60 (0.47-0.76) | 0.90 (0.68-1.17) |
| ORs (95%CI) for service attendance within strata of religious affiliation |  | 0.67 (0.52-0.85) |  | 0.48 (0.37-0.62) |  |
| Measure of effect modification on additive scale: RERI (95%CI) = -0.32 (-0.68, 0.03); p =0.07.  The multiplicative interaction and its 95% CI=0.66 (0.46, 0.94). P value for multiplicative interaction= 0.02 | | | | | |
| Subsequent divorce or separation | | | | | |
| Never or < once/week |  | 1.0 |  | 1.29 (1.04-1.58) | 1.29 (1.04-1.58) |
| > once/week |  | 0.62 (0.50-0.77) |  | 0.65 (0.53-0.80) | 1.04 (0.83-1.32) |
| ORs (95%CI) for service attendance within strata of religious affiliation |  | 0.62 (0.50-0.77) |  | 0.48 (0.38-0.62) |  |
| Measure of effect modification on additive scale: RERI (95%CI) = -0.27 (-0.58, 0.04); p =0.08.  The multiplicative interaction and its 95% CI=0.75 (0.55, 1.01). P value for multiplicative interaction= 0.06 | | | | | |

CI: confidence interval

OR: odds ratio

Multivariable logistic regression model adjusted for age (continuous), calendar year, questionnaire cycle, alcohol consumption (none, 0.1-4.9, 5.0-14.9, ≥15.0 g/d), husband’s education (less than high school, some high school, high school graduate, college, graduate school), good physical or function (yes, no), median family income(dollars/year), geographic region (north, south, middle, other) and religious service attendance in 1992 (never, < 1/week, > 1/week), unemployed in the past two years (yes, no), baseline depression (yes, no), parity (nulliparous, 1-2, 3-4, 5+), prior history of divorce (yes, no), physical exercise (metabolic equivalent values; quintiles), hypertension (yes, no), hypercholesterolemia (yes, no), type 2 diabetes (yes, no), menopausal status (premenopausal, postmenopausal) and postmenopausal hormone use (never, past and current), physical exam in the past 2 years (no , yes for symptoms and yes for screenings), healthy eating score (quintiles), smoking status (never, former, current), pack-years (<10, 10-19, 20-39, ≥40 for former smokers; <25, 25-44, 45-64, ≥65 for current smokers), and BMI (kg/m^2^; <21, 21-22.9, 23-24.9, 25-27.4, 27.5-29.9, 30-34.9, ≥35).
