# Supplementary material for: Religious service attendance, divorce, and remarriage among U.S. nurses in mid and late life
Source: PLoS One. 2018 Dec 3;13(12):e0207778. doi: 10.1371/journal.pone.0207778 (PMC6277070; doi:10.1371/journal.pone.0207778)
Supplement: S4 Table — (DOCX) [file pone.0207778.s004.docx]

S4 Table. Multivariate adjusted association between religious services attendance and subsequent remarriage in the Nurses’ Health Study, 1996-2010

|  | Religious service attendance in 1996 | | | |  |
| --- | --- | --- | --- | --- | --- |
|  | Never | Less than once/week | Once/week | More than once/week | P trend |
| Among widowed women in 1996 |  |  |  |  |  |
| Remarriage cases No. =695 | 116 | 118 | 294 | 167 |  |
| Age-adjusted OR (95% CI) | 1.00 (ref) | 1.56 (1.20-2.02) | 1.42 (1.15-1.77) | 1.53 (1.20-1.94) | 0.002 |
| Multivariable OR (95% CI)^*^ | 1.00 (ref) | 1.48 (1.10-1.97) | 1.39 (1.08-1.77) | 1.49 (1.13-1.97) | 0.01 |
|  |  |  |  |  |  |
| Among divorced women in 1996 |  |  |  |  |  |
| Remarriage cases No. =723 | 247 | 184 | 215 | 77 |  |
| Age-adjusted OR (95% CI) | 1.00 (ref) | 1.39 (1.13-1.70) | 1.14 (0.94-1.39) | 0.97 (0.74-1.27) | 0.04 |
| Multivariable OR (95% CI)^*^ | 1.00 (ref) | 1.23 (0.97-1.56) | 1.09 (0.86-1.37) | 1.12 (0.81-1.54) | 0.42 |
|  |  |  |  |  |  |
| Among women who had separation in 1996 |  |  |  |  |  |
| Remarriage cases No. =197 | 70 | 44 | 58 | 25 |  |
| Age-adjusted OR (95% CI) | 1.00 (ref) | 1.05 (0.69-1.62) | 0.84 (0.57 -1.23) | 0.80 (0.48-1.33) | 0.07 |
| Multivariable OR (95% CI)^*^ | 1.00 (ref) | 1.05 (0.61-1.83) | 0.78 (0.47-1.28) | 0.85 (0.46-1.57) | 0.37 |
|  |  |  |  |  |  |
| Among women who had previously married and self-reported as unmarried in 1996 |  |  |  |  |  |
| Remarriage cases No. =1567 | 423 | 334 | 551 | 259 |  |
| Age-adjusted OR (95% CI) | 1.00 (ref) | 1.30 (1.12-1.50) | 0.92 (0.80-1.04) | 0.84 (0.72-0.99) | 0.002 |
| Multivariable OR (95% CI)^*^ | 1.00 (ref) | 1.22 (1.03-1.45) | 1.04 (0.89-1.21) | 1.09 (0.90-1.32) | 0.66 |

CI: confidence interval

OR: odds ratio

* Multivariable logistic regression model adjusted for age (continuous), calendar year, questionnaire cycle, alcohol consumption (none, 0.1-4.9, 5.0-14.9, ≥15.0 g/d), husband’s education (less than high school, some high school, high school graduate, college, graduate school), good physical or function (yes, no), median family income(dollars/year), geographic region (north, south, middle, other) and religious service attendance in 1992 (never, < 1/week, > 1/week), unemployed in the past two years (yes, no), baseline depression (yes, no), parity (nulliparous, 1-2, 3-4, 5+), prior history of divorce (yes, no), physical exercise (metabolic equivalent values; quintiles), hypertension (yes, no), hypercholesterolemia (yes, no), type 2 diabetes (yes, no), menopausal status (premenopausal, postmenopausal) and postmenopausal hormone use (never, past and current), physical exam in the past 2 years (no , yes for symptoms and yes for screenings), healthy eating score (quintiles), smoking status (never, former, current), pack-years (<10, 10-19, 20-39, ≥40 for former smokers; <25, 25-44, 45-64, ≥65 for current smokers), and BMI (kg/m^2^; <21, 21-22.9, 23-24.9, 25-27.4, 27.5-29.9, 30-34.9, ≥35).
