# Supplementary material for: Religious service attendance, divorce, and remarriage among U.S. nurses in mid and late life
Source: PLoS One. 2018 Dec 3;13(12):e0207778. doi: 10.1371/journal.pone.0207778 (PMC6277070; doi:10.1371/journal.pone.0207778)
Supplement: S5 Table — (DOCX) [file pone.0207778.s005.docx]

S5 Table. Joint effect of religious service attendance in 1996 and religious affiliation on subsequent remarriage

|  | Religious affiliation | | | |  |
| --- | --- | --- | --- | --- | --- |
|  | Protestant | | Catholic | | ORs (95% CI) for religious affiliation within strata of religious service attendance |
| Frequency of religious service attendance |  | OR (95% CI) |  | OR (95% CI) |  |
| Remarriage among widowed women in 1996 | | | | | |
| Never or < once/week |  | 1.0 |  | 0.75 (0.54-1.04) | 0.75 (0.54-1.04) |
| > once/week |  | 1.33 (1.07-1.64) |  | 0.84 (0.67-1.06) | 0.63 (0.52-0.78) |
| ORs (95%CI) for service attendance within strata of religious affiliation |  | 1.33 (1.07-1.64) |  | 1.12 (0.81-1.55) |  |
| Measure of effect modification on additive scale: RERI (95%CI) = -0.27 (-0.67, 0.12); p =0.18.  The multiplicative interaction and its 95% CI=0.86 (0.59, 1.27). P value for multiplicative interaction= 0.46 | | | | | |
|  | | | | | |
| Remarriage among divorced women in 1996 | | | | | |
| Never or < once/week |  | 1.0 |  | 0.97 (0.78-1.21) | 0.97 (0.78-1.21) |
| > once/week |  | 1.16 (0.93-1.43) |  | 0.83 (0.65-1.05) | 0.71 (0.55-0.93) |
| ORs (95%CI) for service attendance within strata of religious affiliation |  | 1.16 (0.93-1.43) |  | 0.85 (0.65-1.10) |  |
| Measure of effect modification on additive scale: RERI (95%CI) = -0.34 (-0.75, 0.07); p =0.10.  The multiplicative interaction and its 95% CI=0.75 (0.54, 1.05). P value for multiplicative interaction= 0.09 | | | | | |
|  |  |  |  |  |  |
| Remarriage among women who were separated in 1996 | | | | | |
| Never or < once/week |  | 1.0 |  | 0.90 (0.61-1.33) | 0.90 (0.61-1.33) |
| > once/week |  | 0.87 (0.57-1.32) |  | 0.78 (0.51-1.18) | 0.90 (0.57-1.41) |
| ORs (95%CI) for service attendance within strata of religious affiliation |  | 0.87 (0.57-1.32) |  | 0.86 (0.55-1.36) |  |
| Measure of effect modification on additive scale: RERI (95%CI) = -0.005 (-0.72, 0.71); p =0.99.  The multiplicative interaction and its 95% CI=0.99 (0.54, 1.81). P value for multiplicative interaction= 0.97 | | | | | |
|  |  |  |  |  |  |
| Remarriage among women who had previously married and self-reported as unmarried in 1996 | | | | | |
| Never or < once/week | 420/6882 | 1.0 |  | 0.91 (0.77-1.08) | 0.91 (0.77-1.08) |
| > once/week | 408/6969 | 1.14 (0.99-1.31) |  | 0.78 (0.67-0.91) | 0.69 (0.59-0.80) |
| ORs (95%CI) for service attendance within strata of religious affiliation |  | 1.14 (0.99-1.31) |  | 0.86 (0.71-1.03) |  |
| Measure of effect modification on additive scale: RERI (95%CI) = -0.29 (-0.55, -0.03); p =0.03.  The multiplicative interaction and its 95% CI=0.76 (0.61, 0.95). P value for multiplicative interaction= 0.02 | | | | | |

CI: confidence interval

OR: odds ratio

Multivariable logistic regression model adjusted for age (continuous), calendar year, questionnaire cycle, alcohol consumption (none, 0.1-4.9, 5.0-14.9, ≥15.0 g/d), husband’s education (less than high school, some high school, high school graduate, college, graduate school), good physical or function (yes, no), median family income(dollars/year), geographic region (north, south, middle, other) and religious service attendance in 1992 (never, < 1/week, > 1/week), unemployed in the past two years (yes, no), baseline depression (yes, no), parity (nulliparous, 1-2, 3-4, 5+), prior history of divorce (yes, no), physical exercise (metabolic equivalent values; quintiles), hypertension (yes, no), hypercholesterolemia (yes, no), type 2 diabetes (yes, no), menopausal status (premenopausal, postmenopausal) and postmenopausal hormone use (never, past and current), physical exam in the past 2 years (no , yes for symptoms and yes for screenings), healthy eating score (quintiles), smoking status (never, former, current), pack-years (<10, 10-19, 20-39, ≥40 for former smokers; <25, 25-44, 45-64, ≥65 for current smokers), and BMI (kg/m^2^; <21, 21-22.9, 23-24.9, 25-27.4, 27.5-29.9, 30-34.9, ≥35).
